# Cinnamon-Derived Compounds Reduce PD-L1 Expression in UV-Exposed Human Skin Cell Line

**DOI:** 10.3390/medicines13020020

**Published:** 2026-06-20

**Authors:** Chidambaram Ramanathan, Richard J. Bloomer, Gus Romero

**Affiliations:** 1 Center for Nutraceutical and Dietary Supplement Research, College of Health Sciences, University of Memphis, Memphis, TN 38152, USA; rbloomer@memphis.edu; 2 GTS Biotech LLC, 4935 Main Street, Suite 7, #334, Spring Hill, TN 37174, USA; gus@gtsbiotech.com

**Keywords:** UVAB radiation, PD-L1, keratinocytes, CTB-1, CTD-1, cinnamon extract, DNA damage

## Abstract

**Background/Objective:** Ultraviolet A and B (UVAB) radiation is a major environmental factor that induces DNA damage and upregulates programmed death-ligand 1 (PD-L1) expression in skin cells, thereby contributing to immune evasion and impaired tissue repair. This study evaluated the protective effects of two purified compounds, Cinnamtannin B1 (CTB-1) and Cinnamtannin D1 (CTD-1), as well as cinnamon extract, in UVAB-irradiated human keratinocyte HaCaT cells. **Methods:** HaCaT cells were exposed to low (20 kJ/m^2^ UVA, 1.3 kJ/m^2^ UVB), medium (30 kJ/m^2^ UVA, 2 kJ/m^2^ UVB), and high (40 kJ/m^2^ UVA, 2.7 kJ/m^2^ UVB) UVAB doses of UVAB radiation. Dose-dependent effects of CTB-1 and CTD-1 (0, 5, 10, 25, and 50 µg/ mL) and cinnamon extract (0, 5, 10, 50, and 100 µg/mL), as well as time-dependent effects (12, 24, and 72 h), were evaluated by measuring PD-L1 expression, cell viability, and DNA damage. **Results:** CTD-1 was the most effective compound, significantly reducing UVAB-induced PD-L1 expression and DNA double-strand breaks without compromising cell viability. CTB-1 also demonstrated protective effects at specific doses and time points; however, higher concentrations reduced cell viability. Cinnamon extract was protective at low concentrations but cytotoxic at higher doses. **Conclusions:** CTD-1, CTB-1, and cinnamon extract attenuated UVAB-induced cellular damage in HaCaT cells, with CTD-1 demonstrating the most favorable protective profile. These findings support the potential of cinnamon-derived compounds as therapeutic candidates for preventing UVAB-induced skin damage and immune dysregulation.

## 1. Introduction

Medicinal plants and their extracts have been used across cultures for therapeutic purposes and represent valuable natural resources. Herbal medicines continue to support healthcare systems by contributing to the treatment of diverse human ailments, and plant-derived compounds have long been incorporated into conventional therapies across many ethnic traditions [1]. Interest in natural products has increased, in part, due to adverse side effects associated with some synthetic chemical treatments [2]. Bioactive compounds found in medicinal plants are rich sources of antioxidants and hold promise for the development of novel therapies, as they can enhance immune function, improve nutrient absorption, and maintain intestinal integrity [3].

Cinnamon is widely known as a culinary spice and is derived primarily from *Cinnamomum cassia*, *C. verum*, *C. burmannii* and *C. loureirori*. Traditionally, cinnamon has been used to treat disorders of the digestive and circulatory systems, arthritis, and the common cold [4]. For example, the use of cinnamon bark to treat rheumatoid arthritis (RA), a chronic inflammatory autoimmune disease, has been described in ancient medicine. Mechanistically, cinnamon bark has been reported to exert anti-arthritic effects through its anti-inflammatory, analgesic, and immune-regulatory properties [5]. The anti-inflammatory activity of cinnamon species has been attributed mainly to procyanidin oligomers present in the bark [6]. Recent studies further suggest that cinnamon bark extract can regulate immune function in vitro and prevent or ameliorate inflammation-associated diseases [2]. Collectively, natural cinnamon compounds with antioxidant and immunomodulatory properties have attracted considerable attention as potential protective agents against ultraviolet A and B (UVAB) radiation-induced skin damage; however, this hypothesis requires direct experimental validation.

Natural compounds in cinnamon primarily consist of cinnamaldehyde and polyphenolic procyanidin oligomers. Cinnamaldehyde is the most abundant component, whereas procyanidin oligomers are present at lower concentrations [7]. Among these procyanidin oligomers are Cinnamtannin B1 (CTB-1) and Cinnamtannin D1 (CTD-1), which are both purified cinnamon-derived compounds currently under investigation for their ability to modulate cellular stress responses [8]. Although cinnamon and its derivatives possess well-characterized antioxidant and anti-inflammatory properties, the effects of CTB-1 and CTD-1 on skin physiology remain poorly defined. We therefore hypothesized that cinnamon extract and its purified components, CTB-1 and CTD-1, may attenuate UVAB-induced PD-L1 expression and DNA damage in keratinocytes.

Ultraviolet (UV) radiation is a major environmental stressor affecting human skin and is a key driver of photoaging, inflammation, immunosuppression, and carcinogenesis [9]. In epidermal keratinocytes, UV exposure generates reactive oxygen species, induces DNA double-strand breaks, and activates cellular stress pathways that upregulate immune checkpoint molecules, including programmed death-ligand 1 (PD-L1) [10]. PD-L1 inhibits T-cell activation and promotes immune tolerance, thereby contributing to immune evasion [11]. Elevated PD-L1 expression on tumor cells and immune cells facilitates immune escape and has been implicated in carcinogenesis and chronic inflammatory disorders [10,11]. In keratinocytes, increased PD-L1 expression has been associated with impaired tissue repair and enhanced susceptibility to malignancy [12].

Recent studies demonstrate that UV-induced PD-L1 expression in keratinocytes occurs independently of immune cell interactions, suggesting an intrinsic cellular stress response mechanism [13]. However, the regulatory pathways governing PD-L1 expression in non-malignant keratinocytes remain poorly understood. Identifying compounds capable of suppressing PD-L1 induction may help preserve skin immune function following UV exposure and potentially reduce the risk of photocarcinogenesis.

Despite growing interest in plant-derived antioxidants, few studies have systematically examined the effects of cinnamon-derived compounds on PD-L1 expression in human skin cells exposed to UV radiation [14,15,16]. The present study addresses this gap by investigating the dose- and time-dependent effects of CTB-1, CTD-1, and cinnamon extract on PD-L1 expression, cell viability, and DNA damage in HaCaT cells—a human skin keratinocyte cell line.

Using immortalized human HaCaT keratinocytes as a model system, we evaluated the effects of CTB-1, CTD-1, and cinnamon extract on UVAB-induced PD-L1 expression following solar-simulated UVAB irradiation. We further assessed PD-L1 expression, cell viability, PD-L1 membrane localization, and DNA damage, as indicated by γH2AX expression. Our findings identify CTD-1 as a particularly effective modulator of UVAB-induced PD-L1 expression and DNA damage.

## 2. Materials and Methods

### 2.1. Cell Culture and Treatments

HaCaT keratinocytes (Cat. No. 300493) were purchased from Cytion, Cell lines Services LLC (Cytion, Sioux Falls, SD, USA) and cultured in DMEM supplemented with 10% fetal bovine serum (FBS) and 1X penicillin-streptomycin (PS) in a 37 °C and 5% CO_2_ incubator. Cell morphology and growth were monitored regularly. For experiments, 0.7 × 10^6^ cells were seeded into polystyrene T25 flasks (Cat. No. 156367) (Thermo Fisher Scientific, Thermo Fisher Scientific, Waltham, MA, USA) and allowed to reach confluence. The day before UV exposure, cells were serum-deprived overnight, and all experiments were performed using overnight serum-starved cells. One hour before UV exposure, cells from each treatment group were supplemented with various concentrations of the three compounds (CTB-1, CTD-1, and cinnamon extracts) provided by GTS Biotech LLC (Spring Hill, TN, USA), or with DMSO as a control. The cinnamon extract consisted of 90.62% total polyphenols, including 15.89% Type A Polymers, 5.2% CTB-1, 3.9% CTD-1, <1% cinnamaldehyde, 0.58% coumarin, <5% moisture, and the remaining <10% consisted primarily of carbohydrates and other naturally occurring constituents. Cells were incubated for 1 h, washed, and transferred into DPBS containing 0.05% magnesium and calcium. Cells were then exposed to solar-simulated UV radiation at low-UV (20 kJ/m^2^ UVA, 1.3 kJ/m^2^ UVB), medium UV (30 kJ/m^2^ UVA, 2 kJ/m^2^ UVB), and high-UV (40 kJ/m^2^ UVA, 2.7 kJ/m^2^ UVB) doses using solar-simulated light (SSL) lamps (UVA340) from Q-Lab corporation (Westlake, OH, USA). UVA340 lamps emit both UVA and UVB in a ratio similar to natural sunlight (93% UVA and 7% UVB). For white-light control, cells were exposed to fluorescent white light (WL) for the same duration as the UVAB exposure. After UVAB and WL exposure, cells were rinsed again with DPBS before being placed back into complete DMEM and post-treated with the same concentration of DMSO, CTB-1, or CTD-1 or cinnamon extract for 24, 48, and 72 h, depending on the experimental conditions. Because each experiment used different compound concentrations and treatment durations, the specific conditions are indicated in the corresponding figure legends.

### 2.2. Western Blot Analysis

The media were aspirated, and the cells were rinsed with PBS. Cells were lysed in RIPA (Cat # 89900) (Thermo Fisher Scientific) buffer supplemented with protease and phosphatase inhibitors and PMSF. The RIPA cell lysis buffer was added to the T25 flasks, and the T25 flasks were incubated on ice for 30 min. Using a cell scraper, the cell lysate was collected, and protein concentration was measured using the BCA assay. A measure of 10–20 μg of protein lysate was loaded onto an 8% acrylamide gel (SDS-PAGE) to resolve the proteins. The proteins were transferred to nitrocellulose membranes, and ponceau staining was performed to confirm protein transfer, followed by blocking with 5% blocking buffer. Primary antibodies for GAPDH (1:1000) (Cat. No. 14C10) (Cell Signaling, Danvers, MA, USA), PD-L1 (1:500) (Cat. No. E1L3N, Rabbit mAb #13684) (Cell Signaling), and γH2AX, a marker of DNA double-strand breaks due to UV (Cat. No. Phospho-γH2AX (Ser139) Antibody #2577 (Cell Signaling), were used and incubated overnight at 4 °C on a shaker. The next day, the membranes were rinsed with 1X TBST three times and then incubated with Anti-rabbit IgG, HRP-linked secondary antibody (1:2000) (Cat. No. 7074) (Cell Signaling) for 1 h at room temperature. After incubation, the membranes were washed three times with 1X TBST. An ECL substrate was used to visualize band intensity, and the band image was captured using an imaging system (iBright 1500, Thermo Fisher Scientific).

### 2.3. WB Band Intensity Measurement

ImageJ software (Version 1.54g) was used to measure band intensity. A rectangular box was drawn, and the same-sized box was used to measure the intensity of each band for GAPDH or PD-L1 expression in the samples. For background subtraction, three identical boxes were placed in three different locations, and background values were obtained to subtract from the experimental intensity values.

### 2.4. Flow Cytometry Analysis

At 24 and 48 h after UVAB exposure with or without CTB-1 or CTD-1, cells were collected by trypsinization. The cell numbers were determined using a hemocytometer. A total of 10,000 cells were washed with PBS and incubated with either FITC-conjugated anti-human PD-L1 antibody (Cat. No. 393606) (Biolegend, San Diego, CA, USA) or FITC-conjugated mouse IgG1κ isotype control (Cat # 400110) (Biolegend). For each sample, 5 µL of antibody was added to 95 µL of staining solution. The reaction tubes were incubated at room temperature in the dark for 30 min. Following incubation, 1 mL of staining solution was added, and the samples were vortexed briefly to help remove unbound antibody. The reaction tubes were then centrifuged at 400× *g* for 5 min at 20 °C. The supernatant was discarded, and cells were resuspended in 250 μL of staining solution by pipetting up and down 10 times. The stained cells were then analyzed by flow cytometry using an Attune NxT instrument. (Thermo Fisher Scientific, Waltham, MA, USA) An initial dotplot (FSC-A vs. FSC-H) was generated to identify singlets for each sample. From this gate, the FSC-H vs. SSC-H plot was created, and 10,000 events were acquired. Dotplots and histograms were then generated from this population. Cells treated with UV + DMSO were stained with IgG1 isotype control to assess non-specific antibody binding and background fluorescence, ensuring that the observed signal reflected specific PD-L1 expression. PD-L1-FITC staining was used to detect and quantify PD-L1-positive cell populations. Data were presented as histograms and dotplots, with fluorescence intensity plotted against the number of detected cells.

### 2.5. MTT Assay

The assay was performed using 96-well plates according to the manufacturer’s instructions (Cat. No. V13154) (Invitrogen; Eugene, Oregon, USA). Briefly, we seeded the HaCaT cells into 96-well plates. After the cells reached confluence, they were treated with either DMSO, CTB-1, CTD-1, or cinnamon extract, and exposed to UV doses as exactly described in the experiment above. Twenty-four hours after the post-treatment incubation, an MTT assay was performed following the manufacturer’s instructions.

### 2.6. Data Analysis

Data were analyzed using one-way ANOVA and statistical significance was determined at *p* < 0.05. Results are presented as mean ± SD. GraphPad Prism 10 software was used to analyze the data and construct the figures.

## 3. Results

### 3.1. Determines the Effects of CTD-1 and CTB-1 on PD-L1 Expression and Cell Viability in the HaCaT Cells

To determine whether UVAB irradiation increases PD-L1 expression, serum-starved HaCaT cells were exposed to a high dose of UVAB (40 kJ/m^2^ UVA and 2.7 kJ/m^2^ UVB).

**Figure 1:**
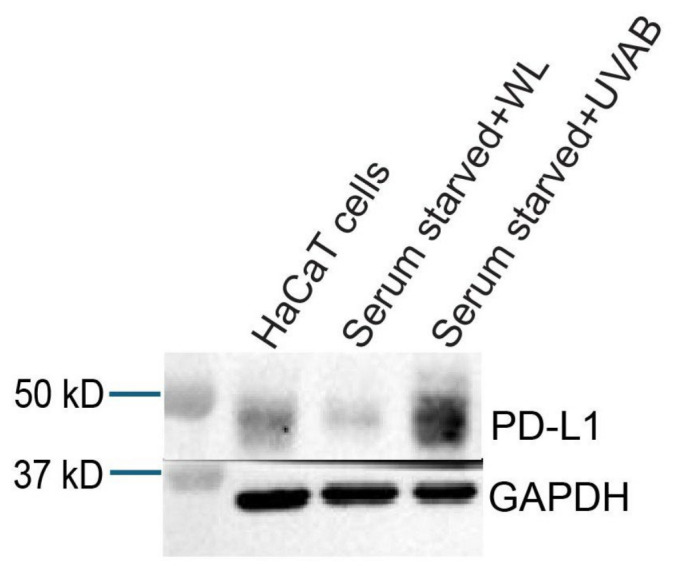
Western blot analysis of PD-L1 expression in HaCaT cells exposed to high-dose UVAB radiation. The overnight serum-starved cells were treated with white light (WL) and high-dose UVAB. HaCAT cells alone, without serum starvation or light exposure, served as the control. Cells from three flasks from each condition have been pooled, and the total protein has been resolved. Blot images show band intensities of PD-L1 and GAPDH expression in the pooled samples.

High-dose UVAB irradiation increased PD-L1 expression compared with overnight serum-starved, white light-exposed, and non-serum-starved control cells (Figure 1). These results validate our experimental conditions and demonstrate that UVAB irradiation induces PD-L1 expression in HaCaT keratinocytes.

**Figure 2:**
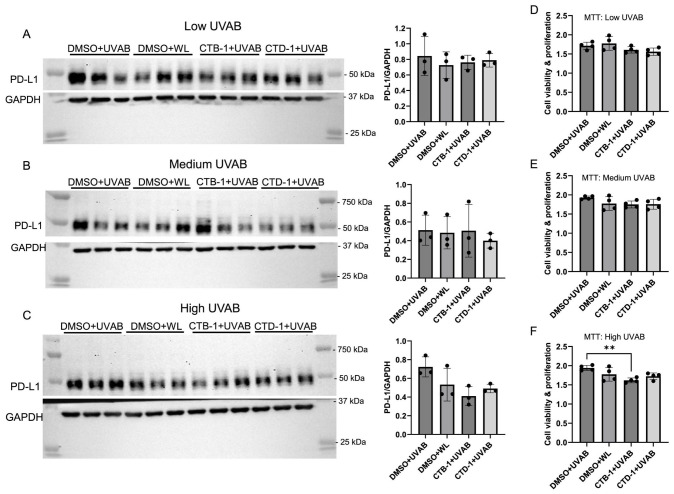
Western blot analysis of PD-L1 expression in HaCaT cells exposed to low, medium, and high doses of UVAB radiation. Cells were treated with DMSO (UVAB control), DMSO (white light control), CTB-1 (10 µg/mL + UVAB), or CTD-1 (10 µg/mL + UVAB). Representative WB blot images are shown (**A**–**C**), and quantified band intensities normalized to GAPDH are presented (**A**–**C**). No significant differences were observed among groups in low (F_3,8_ = 0.2693; *p* = 0.8459) or medium (F_3,8_ = 0.2289; *p* = 0.8737), but there was a trend toward reduced PD-L1 expression with CTB-1 and CTD-1 treatment in the high UVAB dose group (F_3,8_ = 3.833; *p* = 0.0571). MTT assay was used to measure cell viability after different dosages of UVAB exposure. For a low UVAB dose (F_3,12_ = 2.691; *p* = 0.0932) and medium UVAB dose (F_3,12_ = 1.979; *p* = 0.1710), no significant differences were observed across treatment groups (**D**,**E**). For high UV doses, one-way ANOVA indicated a significant difference between groups (F_3,12_ = 6.9131; *p* = 0.0090). Post hoc Tukey’s multiple comparison tests show that the DMSO+UVAB group had higher cell viability compared to the CTB-1+UVAB group (** *p* = 0.058) (**F**).

Next, we tested whether a single dose of cinnamon compounds CTB-1 and CTD-1 could reduce UVAB-induced PD-L1 expression across three UVAB irradiation doses (low, medium, and high) (Figure 2). Four experimental conditions were included at each UVAB dose: DMSO + UVAB, DMSO + white light (WL), CTB-1 (10 µg/mL) + UVAB, and CTD-1 (10 µg/mL) + UVAB. UVAB exposure did not increase PD-L1 expression in a dose-dependent manner, and no statistically significant differences were observed among treatment groups at each irradiation dose (Figure 2). However, at the high UVAB dose, a strong trend was observed between the DMSO + UVAB and CTB-1+ UVAB and CTD-1 + UVAB groups, suggesting a potential protective effect of CTB-1 and CTD-1 under conditions of increased UV stress (F_3,8_ = 3.833; *p* = 0.0571) (Figure 2C). Although this effect did not reach statistical significance, the results suggest that CTB-1 treatment may confer protection against high-dose UVAB-induced PD-L1 expression. Cell viability did not vary much after UVAB exposure for all doses (Figure 2D–F). However, CTB treatment (10 μg/mL) decreased cell viability after a high dose of UVAB exposure. The exact mechanism underlying this decrease is unknown and requires further investigation.

### 3.2. CTB-1, CTD-1, and Cinnamon Extract Reduce UVAB-Induced PD-L1 Expression in a Dose- and Time-Dependent Manner

**Figure 3:**
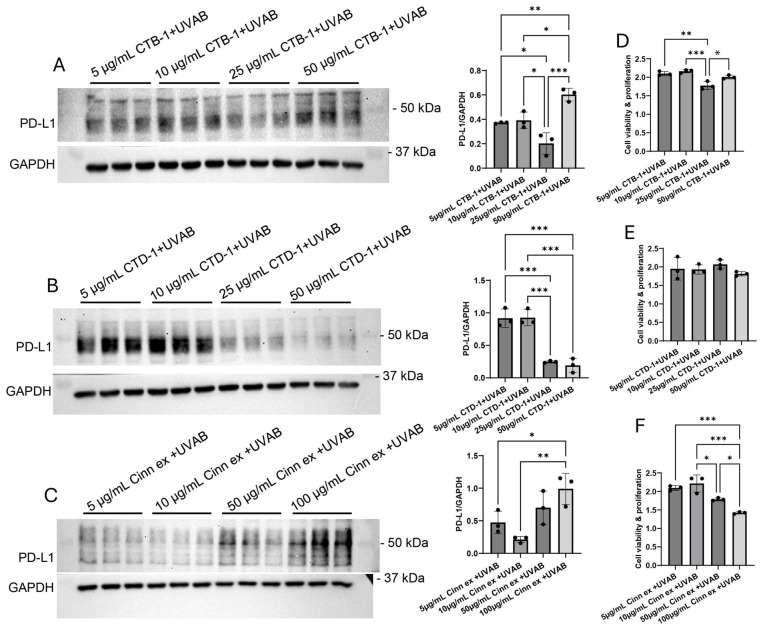
Dose-dependent effects of CTB-1, CTD-1, and cinnamon extract on PD-L1 expression in HaCaT cells exposed to high-dose UV radiation. Representative immunoblots and quantified band intensities are shown for CTB-1 (**A**), CTD-1 (**B**), and cinnamon extract (**C**). The control samples were not included because no differences were observed between the control and the CTD-1 or CTB-1 treatments at any UVAB doses in Figure 2. A significant reduction in PD-L1 expression was observed with CTB-1 at 25 µg/mL (F_3,8_ = 21.24; *p* = 0.0004), CTD-1 at 25 and 50 µg/mL (F_3,8_ = 41.56; *p* = 0.0001), and cinnamon extract at 5–10 µg/mL (F_3,8_ = 8.529; *p* = 0.0071). The MTT cell viability assay was performed with three compounds at four doses: CTB-1 at 5, 10, 25, and 50 µg/mL (**D**); CTD-1 at 5, 10, 25, and 50 µg/mL (**E**); and Cinnamon extract at 5, 10, 50, and 100 µg/mL (**F**) with high UVAB exposure. One-way ANOVA showed that there were significant differences across treatment groups for the CTB-1 compound (F_3,8_ = 17.77; *p* = 0.0007) and the Cinnamon extract compound (F_3,8_ = 24.71; *p* = 0.0002). For the CTD-1 compound, one-way ANOVA indicated no significant difference between groups (F_3,8_ = 1.020; *p* = 0.4333). Post hoc Tukey’s multiple comparison tests reveal significant differences among the means of each group (* *p* = 0.01, ** *p* = 0.001, *** *p* = 0.0001). For CTB-1, the 25 µg/mL dose showed less viability compared with the other groups. For the Cinnamon extract compound, 50 and 100 µg/mL showed lower viability compared to the other groups.

CTB-1/CTD-1 compounds are the main constituents of the cinnamon extract, and both have been used for various physiological functions [8]. To assess their ability to suppress UVAB-induced PD-L1 expression, CTB-1, CTD-1, and a cinnamon powder extract were tested at multiple concentrations following high-dose UVAB exposure. Based on the results of the initial experiment, a high UVAB dose was selected, as it produced the most robust PD-L1 induction and revealed potential compound-dependent effects. CTB-1 reduced PD-L1 expression in a dose-dependent manner at concentrations ranging from 10 to 25 µg/mL (Figure 3A). However, cell viability was also lower at the same dose (25 µg/mL) compared with other doses. These data indicated that 25 μg/mL is the optimal dosage for the protective effects after UVAB exposure. This protective effect may explain the increasing apoptosis of damaged cells. However, higher CTB-1 dosages, such as 50 µg/mL, could be harmful (Figure 3A). CTD-1 was effective at concentrations of 25–50 µg/mL, significantly reducing PD-L1 expression (Figure 3B). Cell viability at different doses of CTD-1 was not affected. These data indicate that CTD-1 dosages above 25 µg/mL should be used to achieve protective effects on PD-L1 levels after UVAB exposure (Figure 3E). Cinnamon powder extract reduced PD-L1 expression at 10 µg/mL but caused toxicity at higher concentrations (Figure 3C). The viability of the cells was not affected at the lower two doses, but it was affected at higher doses of Cinnamon extract (50 and 100 µg/mL) (Figure 3F). The results suggest a dose-dependent reduction of PD-L1 expression in vitro.

**Figure 4:**
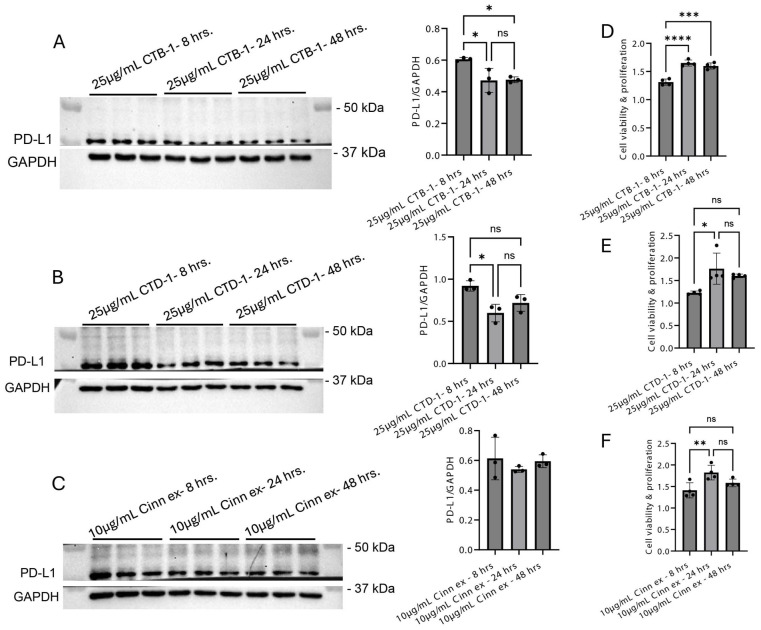
Time-dependent effects of CTB-1 (25 µg/mL), CTD-1 (25 µg/mL), and cinnamon extract (10 µg/mL) on PD-L1 expression following high-dose UV exposure are shown in (**A,B,C**), respectively. Since no differences were observed between the control and CTD-1 or CTB-1 treatments across UVAB doses in Figure 2, the control samples were not included. CTB-1 treatment significantly reduced PD-L1 expression at 24 and 48 h compared with 8 h (F_2,6_ = 8.413; *p* = 0.0182). CTD-1 treatment significantly decreased PD-L1 expression at 24 h compared with 8 h (F_2,6_ = 9.659; *p* = 0.0133). In contrast, cinnamon extract did not show significant differences across time points (F_2,6_ = 0.5740; *p* = 0.5914). The MTT cell viability assay was performed using three compounds—CTB-1 (25 µg/mL), CTD-1 (25 µg/mL), and Cinnamon extract (10 µg/mL)—each tested at a single dose in combination with high-dose UV exposure in a time-dependent manner. For CTB-1, one-way ANOVA revealed significant differences among time points (F_2,9_ = 44.92; *p* = 0.0001) (**D**). Post hoc analysis showed that cell viability was significantly higher at 24 and 48 h compared with 8 h (*** *p* = 0.0001; **** *p* < 0.0001). For CTD-1, one-way ANOVA also revealed significant differences among groups (F_2,9_ = 7.393; *p* = 0.0126) (**E**). Post hoc comparison reveals that cell viabilities are significantly higher in 24 h of treatment compared with 8 and 48 h (* *p* = 0.01). For Cinnamon extract treatment, one-way ANOVA showed significant differences between groups (F_2,9_ = 7.673; *p* = 0.0114) (**F**), with post hoc analysis revealing that cell viability was significantly higher at 24 h compared with both 8 and 48 h. (** *p* = 0.001). ns: not significant.

To evaluate time-dependent effects, a single effective dose of each compound (CTB-1, CTD-1, and cinnamon extract) was selected and tested following high-dose UVAB irradiation. Both CTB-1 (25 µg/mL) and CTD-1 (25 µg/mL) significantly reduced PD-L1 expression after 24 h of treatment. These effects were maintained at 48 h but were not observed at 8 h (Figure 4A,B). No significant time-dependent effect was observed for the cinnamon extract (10 µg/mL) (Figure 4C). MTT assay results for CTB-1 showed higher cell viability at 24 and 48 h as compared to 8 h of treatment. These findings suggest that CTB-1 at 25 µg/mL should be maintained for at least 24 h to reduce UV-induced PD-L1 expression (Figure 4D) effectively. Similarly, MTT assay results for CTD-1 showed higher cell viability at 24 and 48 h than at 8 h, indicating that 25 µg/mL CTD-1 is an optimal concentration for reducing UV-induced PD-L1 expression during a 24 h treatment period (Figure 4E). Overall, these results suggest that CTB-1 and CTD-1 reduce UVAB-induced PD-L1 expression within a 24–48 h treatment window.

### 3.3. Determination of PD-L1 Localization in HaCaT Cells

**Figure 5:**
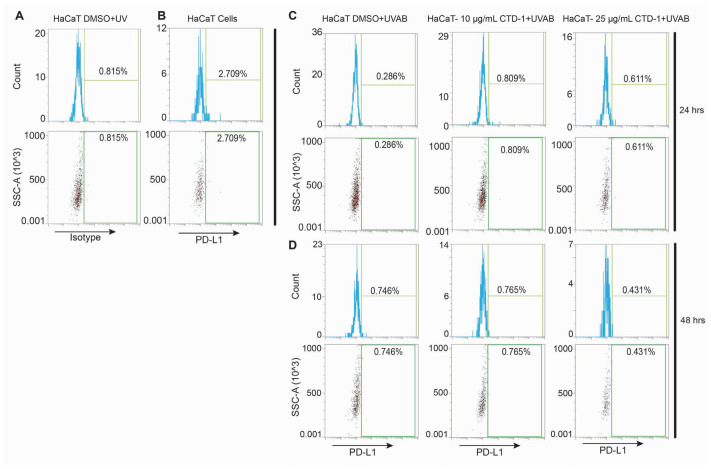
Flow Cytometry Analysis of PD-L1 Surface Expression in HaCaT Cells. (**A**) DMSO+UVA+B-treated cells were stained with the isotype control. (**B**) Untreated HaCaT cells were stained with the FITC-conjugated anti-human PD-L1 antibody. Both A and B cells were analyzed after 24 h. (**C**,**D**) HaCaT cells treated with DMSO+UV, CTD-1 (10 µg/mL), or CTD-1 (25 µg/mL) were stained with FITC-conjugated anti-human PD-L1 antibody and analyzed after 24 and 48 h. Blue histograms and dotplots represent PD-L1-negative populations, while green rectangles highlight PD-L1-positive regions. Representative histograms and dotplots are shown. The results demonstrate that the PD-L1 protein localized to the cell membrane was not detected under any of the experimental conditions.

The objective of this experiment was to determine whether PD-L1 localizes to the cell membrane or remains intracellular in HaCaT cells. Flow cytometry analysis revealed no detectable membrane-bound PD-L1 expression (Figure 5A,B). Instead, PD-L1 was predominantly intracellular under both untreated and UVAB-irradiated conditions (Figure 1, Figure 2, Figure 3 and Figure 4). To further determine the effect of the cinnamon-derived compound CTD-1 on PD-L1 localization, HaCaT cells were treated with DMSO+UVAB, CTD-1 (10 µg/mL), or CTD-1 (25 µg/mL), stained with a FITC-conjugated anti-human PD-L1 antibody, and analyzed at 24 and 48 h. Consistent with earlier observations, no membrane-associated PD-L1 expression was detected under any treatment condition (Figure 5A–D), indicating that PD-L1 remains intracellular in HaCaT cells even following UVAB exposure.

### 3.4. Effect of CTD-1 on UVAB-Induced DNA Damage

**Figure 6:**
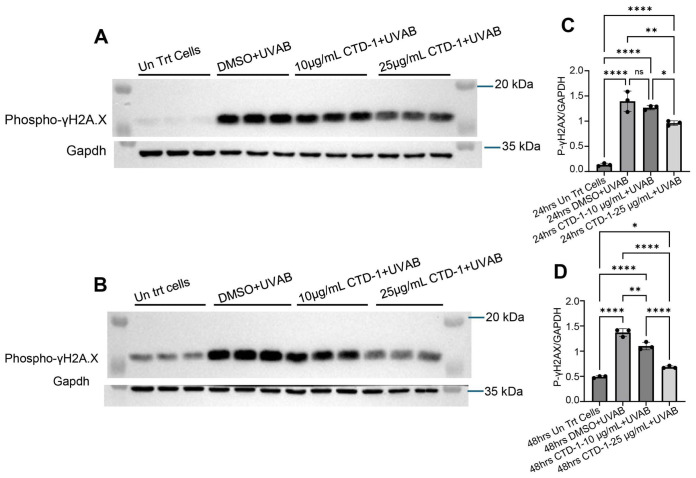
Untreated cells and cells treated with either DMSO or CTD-1 (10 µg/mL; 25 µg/mL), combined with high UV exposure, were analyzed for p-γH2AX protein expression in HaCaT cells at 24 and 48 hrs. Raw Western blot results are shown in panels (**A**,**B**), and the corresponding quantified band intensities are presented in panels (**C**,**D**). (**A**,**C**) At 24 hrs, one-way ANOVA revealed significant differences between groups (F _3, 8_ = 83.90, *P* = 0.0001). Cells exposed to high UV and treated with DMSO expressed significantly higher levels of p-γH2AX protein compared with untreated cells. Treatment with 25 µg/mL CTD-1 significantly reduced UV-induced p-γH2AX expression compared with both the UV + DMSO control and the 10 µg/mL CTD-1-treated groups (* *p* = 0.01; ** *p* = 0.001; **** *p* < 0.0001). (**B**,**D**) At 48 hrs, one-way ANOVA revealed significant differences between groups (F_3,8_ = 154.7, *p* = 0.0001). Cells exposed to high UV and treated with DMSO expressed significantly higher levels of p-γH2AX protein compared with untreated cells. Treatment with either 10 µg/mL or 25 µg/mL CTD-1 significantly reduced UV-induced p-γH2AX expression compared with the UV + DMSO control group. Furthermore, 25 µg/mL CTD-1 was significantly more effective than 10 µg/mL CTD-1 in reducing p-γH2AX expression (* *p* = 0.01; ** *p* = 0.001; **** *p* < 0.0001). These findings indicate that high UV exposure induces p-γH2AX, a marker of DNA double-strand breaks, and that CTD-1 treatment at both doses significantly attenuates the UV-induced p-γH2AX expression after 48 h. ns: not significant.

Because CTD-1 significantly reduced UVAB-induced PD-L1 expression in a dose- and time-dependent manner (Figure 3 and Figure 4), we next examined whether CTD-1 could attenuate UVAB-induced DNA damage. DNA double-strand breaks were assessed by measuring phosphorylated γH2AX (p-γH2AX) expression at 24 and 48 h following high-dose UVAB exposure. As expected, p-γH2AX expression, a marker of UV-induced DNA double-strand breaks, was significantly increased following UVAB irradiation at both time points. Treatment with CTD-1 (25 µg/mL) markedly reduced UVAB-induced p-γH2AX expression at 24 h (Figure 6A,C). At 48 h, both 10 and 25 µg/mL doses of CTD-1 significantly attenuated UVAB-induced p-γH2AX expression (Figure 6B,D). These results indicate that CTD-1 mitigates UVAB-induced DNA damage in HaCaT keratinocytes.

## 4. Discussion

For centuries, cinnamon has been used as a culinary spice, as traditional medicine, and as an adjunctive therapy for metabolic syndrome (MetS) and related disorders [17]. In recent years, growing evidence has supported its therapeutic efficacy in dyslipidemia, hypertension, polycystic ovary syndrome, and inflammatory conditions. Dyslipidemia, characterized by imbalanced triglyceride and cholesterol levels, is a major contributor to metabolic and cardiovascular disease [1,3,16,17,18]. Cinnamon exhibits anti-thrombotic, antispasmodic, anti-ulcerogenic, anti-allergic, anti-inflammatory, antidiabetic, and antihypertriglyceridemic activities—largely attributed to its phytochemical composition, including procyanidin oligomers. Clinical studies have further demonstrated the utility of cinnamon’s anti-inflammatory properties in managing diabetes and related metabolic disorders [19,20].

The procyanidin oligomers from cinnamon are widely used for their antioxidant and anti-inflammatory effects; however, little is known about the molecular mechanisms underlying their mode of action. A previous study demonstrated that CTD-1 ameliorated inflammation in dextran sulfate sodium (DSS)-induced colitis in mice. In this study, the mechanisms underlying the anti-inflammatory effect involve activation of the AMPK and mTOR pathways by CTD-1, which helps restore the balance between regulatory T (Treg) and effector T cells, a balance often disrupted in cancer [21]. Similarly, another study demonstrates that CTB-1 delays the development of osteosarcoma by regulating the miR-128/peptidylprolyl isomerase F (PPIF) pathway [22]. Together, these findings suggest that CTD-1 and CTB-1 suppress cancer cell growth, invasion, and migration while exerting anti-inflammatory and immunomodulatory effects by regulating specific signaling pathways.

Programmed death-ligand 1 (PD-L1) is a type I transmembrane protein that plays a central role in regulating cellular and humoral immune responses. During pathogenic challenge, antigen-specific CD8^+^ cytotoxic T cells and CD4^+^ helper T cells become activated and expand [23]. Engagement of the programmed death-1 (PD-1) receptor with PD-L1 delivers an inhibitory signal that suppresses effector T-cell proliferation while promoting the survival of regulatory T cells. PD-1 is expressed on activated T cells, B cells, and macrophages, whereas PD-L1 is constitutively or inducibly expressed in both hematopoietic and non-hematopoietic cells, enabling broad immune regulation across tissues [24].

Overexpression of PD-L1 on tumor cells is strongly associated with immune evasion and variable clinical outcomes in several cancers. In the skin, PD-L1 expression has been reported in cutaneous squamous cell carcinoma. Because PD-L1 contributes to immune tolerance, inflammation resolution, and immunosuppression, understanding its regulation in keratinocytes under physiological and stress conditions is critical for elucidating early events in skin tumorigenesis [25].

UV radiation is both an inflammatory and genotoxic stressor and represents the primary etiological factor in the development of most skin cancers. Despite its central role in cutaneous carcinogenesis, it remains unclear whether UV exposure directly induces PD-L1 expression in keratinocytes. Determining whether UV radiation activates PD-L1 signaling pathways in epidermal cells is therefore essential for understanding UV-mediated immune modulation. In the present study, we demonstrate that two cinnamon-derived compounds, CTB-1 and CTD-1, exert protective effects on keratinocyte health and UV-induced immunological responses, consistent with previous reports [8]. Cinnamon powder has been widely used to treat nutritionally induced metabolic disorders [26]. Its active component, cinnamaldehyde, has been shown to possess antioxidant and anti-inflammatory properties, including reactive oxygen species (ROS) scavenging and modulation of UV-induced signaling pathways such as NF-kB and MAPK, thereby reducing oxidative stress and cellular damage [16,27]. These properties suggest a potential role for cinnamaldehyde in protecting against UV-induced skin injury.

In our study, cinnamon-derived compounds attenuated UV-induced PD-L1 expression in HaCaT keratinocytes. This is consistent with previous findings that cinnamaldehyde exhibits anticancer properties and modulates pathways involved in tumor growth and progression [27]. Notably, CTB-1 and CTD-1 reduced PD-L1 expression at relatively low concentrations without compromising cell viability, highlighting their therapeutic potential [28]. The current study supports the notion that CTD-1 exhibited protective effects at specific doses and time points but reduced cell viability at higher concentrations, suggesting a mechanism involving the selective elimination of damaged cells via apoptosis.

PD-L1 is classically expressed on the cell surface of the immune cells [29]. In contrast, in our study, we did not detect membrane-localized PD-L1 in HaCaT cells. Flow cytometric analysis revealed that PD-L1 was predominantly intracellular with no detectable plasma membrane expression under either basal or UV-irradiated conditions. These findings suggest that PD-L1 surface expression in keratinocytes may be below the assay’s detection threshold or that PD-L1 is not efficiently trafficked to the cell surface under these experimental conditions. Together, these data indicate that PD-L1 is primarily intracellular in keratinocytes, consistent with their epithelial origin and in contrast to immune and cancer cells, which typically display membrane-localized PD-L1. Although a recent study reported membrane-associated PD-L1 expression in HaCaT cells [30], the absence of representative flow cytometry plots limits direct comparison with our findings. The intracellular localization observed here raises important questions regarding the functional role of PD-L1 in keratinocytes and warrants further investigation.

DNA double-strand breaks (DSBs) represent severe genomic lesions, particularly in proliferating cells. Exogenous sources, such as ionizing radiation, UVA and UVB exposure, and chemical DNA-damaging agents, as well as endogenous sources such as reactive oxygen species (ROS), contribute to replication stress, fork collapse, and genomic instability. UV-induced DNA double-strand breaks increase the phosphorylation of the histone variant H2AX (γH2AX), a well-established marker of DSBs. DNA damage, particularly UVAB-induced DSBs, activates ataxia-telangiectasis mutated kinase (ATM)/ATM and Rad3-related (ATR)/Checkpoint kinase 1 (Chk1) signaling pathways and has been shown in cancer cells to upregulate PD-L1 expression, thereby facilitating immune evasion [31]. This process involves signal transducer and activator of transcription 1 and 3 (STAT1/3) and interferon regulatory factor 1 (IRF1) pathways, in which ATM/ATR-mediated DNA damage response (DDR) signaling promotes PD-L1 transcription via Chk1 activation [31,32]. In addition, p53 acts as a key regulator linking tumor suppression and immune checkpoint control, including PD-L1 expression. The Janus kinase (JAK)/STAT signaling pathway is also a major driver of PD-L1 expression, where phosphorylated STAT1 and STAT3 translocate to the nucleus and bind the PD-L1 promoter to enhance expression [32]. Cinnamon-derived compounds and fragrances have previously been reported to protect skin from UV-induced lesions and photoaging [15,16]. Consistent with these reports, our study demonstrates that CTD-1 significantly reduces UVAB-induced γH2AX phosphorylation, indicating reduced DNA damage. These findings suggest that cinnamon-derived compounds may have potential applications in topical formulations designed to protect against UV-induced skin damage. In addition, UVAB exposure increases ROS production, leading to lipid peroxidation and oxidative DNA lesions, such as 8-oxo-dG, in HaCaT cells. In this context, CTD-1, CTB-1, and cinnamaldehyde may decrease ROS accumulation, reduce 8-oxo-dG formation and lipid peroxidation, lower γH2AX, and ultimately attenuate UV-induced PD-L1 expression.

The findings from the current study align with previous reports describing the immunomodulatory and DNA-protective effects of CTD-1. However, the intracellular localization of PD-L1 in keratinocytes observed here highlights a gap in our understanding of PD-L1 function outside immune and malignant cells. This study is limited in that it does not fully evaluate the protective effects of other cinnamon-derived compounds, such as cinnamon extract and CTB-1, against UVAB-induced DNA damage; these effects remain to be determined. In addition, the in vitro design may not fully capture the complexity of cutaneous immune responses in vivo. Future studies using UV-irradiated animal models will be necessary to validate these findings and to assess the effects of cinnamon-derived compounds on cutaneous immune regulation and inflammation. Further mechanistic studies are also needed to elucidate the signaling pathways through which these compounds exert their protective effects.

## 5. Conclusions

CTD-1, CTB-1, and cinnamon extract exhibit protective effects against UV-induced PD-L1 expression and DNA damage in HaCaT keratinocytes, with CTD-1 demonstrating significant efficacy. These findings support further investigation of cinnamon-derived compounds as potential therapeutic agents for mitigating UV-induced skin damage and immune dysregulation.

## Data Availability

All data generated and analyzed during this study are included in this article. Further inquiries can be directed to the corresponding author.
